# Targeting mitochondrial quality control of T cells: Regulating the immune response in HCC

**DOI:** 10.3389/fonc.2022.993437

**Published:** 2022-09-23

**Authors:** Yixue Xia, Binghong Gao, Xue Zhang

**Affiliations:** ^1^ School of Kinesiology, Shanghai University of Sport, Shanghai, China; ^2^ School of Elite Sport, Shanghai University of Sport, Shanghai, China; ^3^ Shanghai Key Lab of Human Performance, Shanghai University of Sport, Shanghai, China

**Keywords:** mitochondria, immune cell, metabolism, T cells exhaustion, hepatocellular carcinoma (HCC)

## Abstract

Most of the primary hepatocellular carcinoma (HCC) develops from Viral Hepatitis including Hepatitis B virus, Hepatitis C Virus, and Nonalcoholic Steatohepatitis. Herein, T cells play crucial roles combined with chronic inflammation and chronic viral infection. However, T cells are gradually exhausted under chronic antigenic stimulation, which leads to T cell exhaustion in the tumor microenvironment, and the exhaustion is associated with mitochondrial dysfunction in T cells. Meanwhile, mitochondria play a crucial role in altering T cells’ metabolism modes to achieve desirable immunological responses, wherein mitochondria maintain quality control (MQC) and promote metabolism regulation in the microenvironment. Although immune checkpoint inhibitors have been widely used in clinical practice, there are some limitations in the therapeutic effect, thus combining immune checkpoint inhibitors with targeting mitochondrial biogenesis may enhance cellular metabolic adaptation and reverse the exhausted state. At present, several studies on mitochondrial quality control in HCC have been reported, however, there are gaps in the regulation of immune cell function by mitochondrial metabolism, particularly the modulating of T cell immune function. Hence, this review summarizes and discusses existing studies on the effects of MQC on T cell populations in liver diseases induced by HCC, it would be clued by mitochondrial quality control events.

## 1 Introduction

Based on the epidemiology of Hepatocellular Carcinoma (HCC), the main risk factors for HCC are increasingly associated with hepatitis C (HCV) post-sustained virological response, suppressed hepatitis B virus (HBV) on treatment, and non-alcoholic fatty liver disease (NAFLD) (1). One of the essential functions of the liver is metabolism, however, it also works as a lymphoid organ full of immune cells. Once the inter-hepatic microenvironment is disturbed by acute or chronic inflammatory conditions (e.g., HBV infection), the number and localization of these lymphocytes in the liver would be altered (2, 3). Thus, when immune tolerance is triggered, hepatocytes become targets of viral and tumor-induced immune-mediated destruction, thereby leading to autoimmune processes that result in liver injury and even HCC (4, 5). In addition, infection, tumor formation, and autoimmunity in the liver are influenced by immunopathology, in which T cells often play a key role. Immunosuppression predominates in chronic infections (e.g, chronic HBV and chronic HCV infections) as well as in liver cancer (e.g., HCC) (6).

Mitochondria are tremendously regulated to constantly satisfy the energetic demands of the immune cells. The manipulation of mitochondrial mass is a crucial determinant of energy metabolism among the multiple regulatory strategies (7). In general, the events of mitochondrial quality control include biogenesis, fusion, fission, and mitophagy, but mitochondrial transfer also is described as a means of quality control in this review. Herein, mitochondrial biogenesis responds to the demands for the repair and regeneration of defective mitochondria and cellular energy demand for normal functions (8). In addition to controlling the mitochondrial shape, mitochondrial fission and fusion regulate ROS production, calcium homeostasis, and oxidative phosphorylation (9). Similarly, mitophagy serves as the specific degradation mechanism of cellular aged and/or damaged mitochondria (10). Moreover, contrary to the previous description, mitochondrial transfer happens from cell to cell. Recipient cells generally gain favorable metabolism *via* intrinsic regulation of mitochondria (11). Targeting mitochondrial quality control in T cells could be an important perspective for exhausted T cells with dysfunctional mitochondria. Diverse T cell subsets have distinct metabolism demands to facilitate their effector function during the immune response against the pathogen (12). Furthermore, various metabolic processes happen in immune cells to produce adequate amounts of energy for proliferation and create a variety of biosynthetic intermediate. Mitochondria play a vital role in the metabolism reprogramming of T cells for desired immune response. Although there is some research on mitochondria function in immune cells, targeting mitochondrial quality control in T cells may still deserve more attention.

## 2 T cells perform immune functions in liver

Immunotherapy regimens broaden the range of options for the therapy of HCC and other related liver diseases. In particular, T cell-based immunotherapy, in combination with other therapies, has achieved much better results than traditional therapies alone (13). Mitochondria, as the central hub of multiple metabolic pathways, integrate multiple metabolic pathways and contribute to the metabolic reprogramming of T cells. Herein, diverse immunometabolism has respective modes whose precise transformation in metabolic pathways couples to immune effector functions (see **Table 1**). Some studies have demonstrated that senescence is associated with mitochondria in T cells, damaged mitochondria induced by deficiency of mitochondrial transcription factor A (TFAM) works as the accelerator of senescence which causes T cells metabolic disorder and then leads to chronic inflammation (23). The liver consists of five distinct anatomical systems, including the vascular system, hepatic lobule, hepatic sinusoids, the biliary system, and the stroma (2, 23). Circulating T lymphocytes flow through the hepatic sinuses, Naive T cells remain static without stimulation and play corresponding roles with the “cruising” of the blood system to recognize abnormal signals entering the tissue. Wherein tissue-resident memory T lymphocytes enter tissues through homing receptors and remain in a dynamic state. The two mechanisms coordinated with each other active immune monitoring (24). Most of the primary HCC is caused by Virus Hepatitis including Hepatitis B virus (HBV), Hepatitis C virus (HCV), and Nonalcoholic Steatohepatitis (NASH) (25). Immune lymphocytes coexist with cancer cells in the tumor micro-environment, however, cancer cells can better proliferate and escape from immunological surveillance. In addition, patients with HCC who have CD8^+^ T cell infiltration have a reliable prognostic and predictive value, and the absence of CD4^+^ T cell also exacerbates HCC progression (26–28). A poor prognosis was associated with Treg and a good prognosis with CD8^+^ Tm in HCC. Meanwhile, the prognosis of HBV-related HCC is poor because of immunosuppressive and more exhausting than non-viral-related HCC (29). However, in the early stage of hepatocarcinogenesis—chronic liver disease, the excessive response of immune cells is also an important factor that leads to host liver damage and eventually induces hepatocellular carcinogenesis. When it comes to excessive immunity, targeting and enhancing regulatory immune cells is an effective treatment that can be considered. Moreover, the antitumor effect of host CTL was not enough to eliminate HCC. Therefore, immune cell therapy at present needs to enhance the anti-tumor effect in proliferation and differentiation stages, including intervening in the metabolic modes of immune cells in the tumor microenvironment, thus reducing the progress of depleted T cells, and thereby achieving sustained and efficient immune response.

**Table 1:** Diverse T cell subset immunometabolism has distinct modes.

Cell group	Subsets	Immunometabolism modes	Reference
naïve T cell	CD4^+^α/β T cell CD8^+^α/β T cell	Fatty acid oxidation Amino acid metabolism	(14, 15)
			
effector T cell	Th1 Th2 Th9 CTL CD4^+^Treg CD8^+^Treg	Glycolysis Fatty acid synthesis Amino acid metabolism	(16–20)
		TCA cycle	
memory T cell	CD8^+^ memory T cell Tissue-resident memory T cell	TCA cycle Fatty acid oxidation	(21, 22)

## 3 Mitochondrial quality control of T cells on immune response

**Figure 1:**
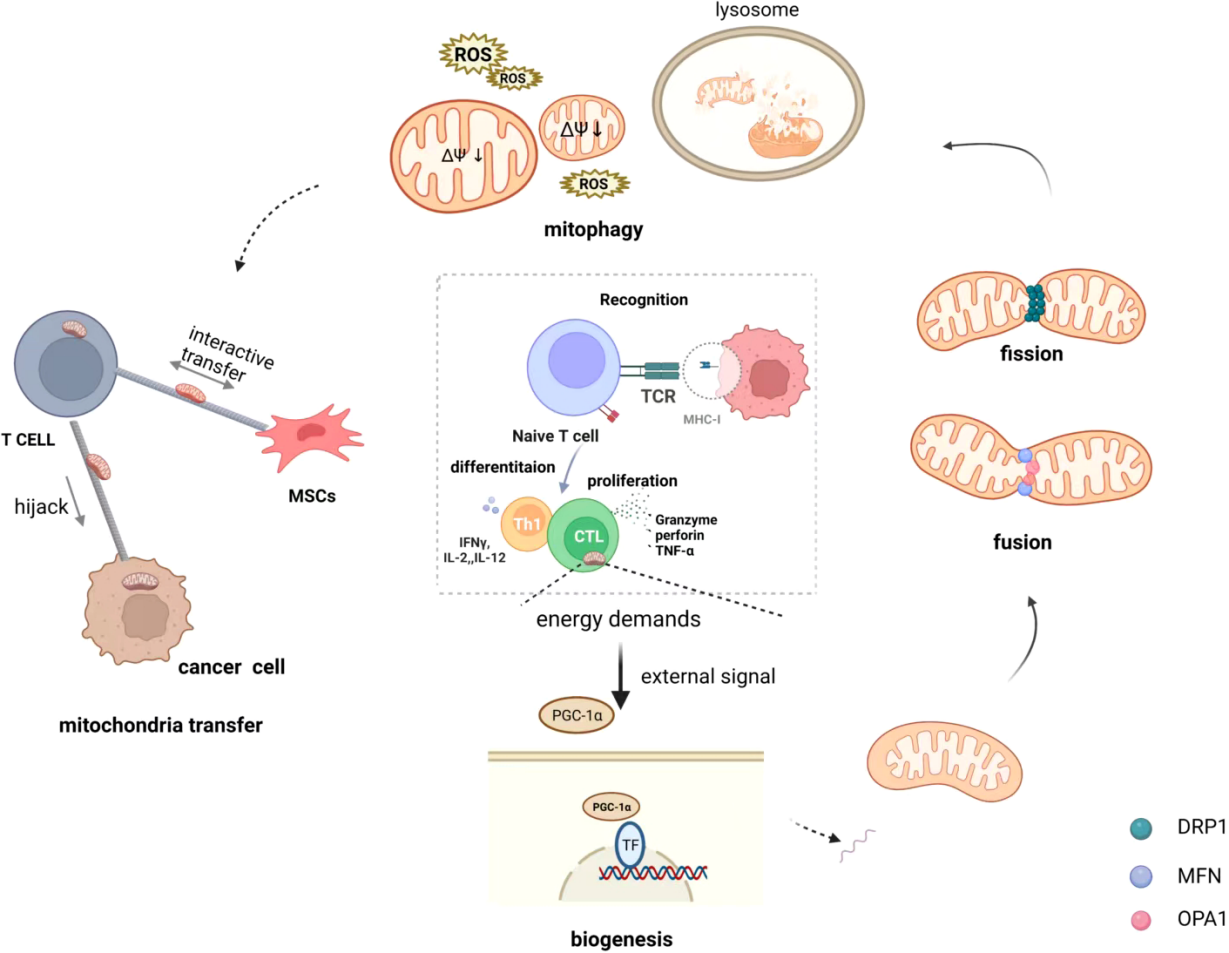
Mitochondrial quality control of T cells on immunometabolism. Immune cell differentiation and function are crucially dependent on specific metabolic programs dictated by mitochondria and the specific processes that occur in mitochondria are intimately linked to their morphology that is shaped by biogenesis, fusion and fission events, and mitophagy. In addition, mitochondria transfer also is regarded as a means of quality control that happens from cell to cell. They receive different signals from external stimuli that dynamically maintain the quality of the mitochondria in the cell to maintain normal energy metabolism. Mitochondrial transfer, in particular, occurs when mitochondria are damaged and unable to maintain normal function, and inevitably affects the mass of intracellular mitochondria.

The host immune system defends against invading pathogens and unfamiliar tumor antigens at any time. Once recognized, immune cells rapidly respond, proliferate, and differentiate (30, 31). In general, T lymphocytes patrol in peripheral blood and immediately rush to the “site” to fight against tumors or pathogens after receiving signals, particularly memory T lymphocytes have immune memory and respond quickly. Mitochondria accumulate at the uropod of T cells and promote phosphorylation and activation of the myosin light chain (catalytic subunit of myosin II) during migration (32). Furthermore, the function of immune cells is heavily dependent on mitochondrial metabolism and is closely linked to mitochondrial morphology, which is affected by events such as mitochondrial biogenesis, fusion, fission, and mitochondrial autophagy, meanwhile, mitochondrial transfer can alter mitochondrial mass (33, 34). (**Figure 1**)

### 3.1 Mitochondrial biogenesis favors the metabolic reprogramming in T cells

In general, T cells play a pivotal role in the antitumor immune response and require a purposeful energy source to raise their cellular functions. Therefore, T cells must have an adequate supply of nutrients and an efficient mechanism to generate ATP (35). As a powerhouse of the cell, mitochondria also play a significant role in the immune responses of T cells. In addition, T cells require enormous energy to synthesize inflammatory factors during the progression of liver diseases such as HBV infection and HCC (36). Meanwhile, mitochondrial biogenesis is a vital part of mitochondrial quality control. T cells apply for an anabolic metabolism phenotype upon engagement of the TCR, thus enabling rapid shifts in resistance from quiescence to activation, and proliferation, thereby increasing the mitochondrial mass for energy demands (37). Fischer et al. verified that mitochondrial biogenesis, proceeding rapidly in nascent activated CD8^+^ T cells, was critical for supporting the production of cytokines by human naïve CD8^+^ T cells in early immune response (38).

#### 3.1.1 PGC-1α—a pivot in mitochondrial biogenesis

It is PGC-1α that highly expressed in skeletal muscle and liver, which belongs to the class of coactivators that can activate the nuclear receptor peroxisome-proliferator-activated receptor γ (39). In addition, PGC-1α is positioned upstream of the mitochondrial biogenesis system, which is the junction between exterior stimulus indicators and the internal regulation of mitochondria. As proven in the preceding work of Partha. S et al. proved that mitochondria were activated by Bezafibrate, an agonist of PGC-1a/PPAR complexes, which prompted the anti-tumor effects of PD-1 blockade. Moreover, it enhanced the proliferation of Naive T cells and CTL function by activating mitochondria, increasing oxidative phosphorylation, and glycolysis (40).

Repetitive antigenic stimulation and persistent infections of cancer cells may cause T cell exhaustion, this nonfunctional state with distinct epigenetic, transcriptomic, and metabolic features (41). Meanwhile, telomere erosion and mitochondrial disruption are prominent features of senescent cells as cellular functions progressively decline, including T cells with anti-tumor immune responses. Impaired mitochondria are the important trigger of T cell exhaustion induced by prolonged antigenic stimulation in chronic infections (42). Schank et al. showed that disruption of telomere integrity leads to T cell senility and apoptosis *via* the telomeric DNA damage response (DDR). The p53-PGC-1α-NRF-1 axis contributed to mitochondrial disorder in the setting of telomeric DDR (43). This study also suggested that focusing on this axis, might offer an alternative and novel strategy to block damaged telomeres from regulating mitochondria and thus causing T cell dysfunction. In addition, adverse signals are produced by metabolic stress in immune cells, particularly persistent antigenic stimulation. Insistent stimulation accelerated Blimp-1-mediated repression of PGC1α-dependent mitochondrial reprogramming, thus triggering cells to poorly respond to hypoxia. Impaired mitochondria produced unbearable levels of ROS, enough to promote exhausted-like status (41). Malinee et al. recently reported that an epigenetic modulator termed EnPGC-1 (BI-PIP) was designed which could enhance the expression of PGC-1 α/β in murine primary CD8^+^ T cells (44). They illustrated that enPGC-1 induced PGC-1α to promote mitochondrial biogenesis and combined with PD-1 blockade enhances the antitumor response and immune metabolism of effector CD8^+^ T cells, thereby suggesting that targeting mitochondrial biogenesis combined with immune checkpoint inhibitors may improve the efficacy of immunotherapy.

Meanwhile, in terms of T effector memory (Tem) cells, T effector responses of CD4^+^Tem cells could be enhanced by platelet factor 4 through Akt-PGC1α-TFAM signals. Mass spectrometric analysis indicates that PF4 regulates CD4^+^ T cell responses by modulating cellular metabolism. Herein, the mechanism by which PF4 acts is through the PF4 receptor CXCR3, which attenuates Akt activity, decreases PGC-1α phosphorylation, elevates PGC-1α function, and increases mitochondrial transcription factor A (TFAM) expression. Whereby TFAM increased mitochondrial biogenesis and subsequently enhanced Th1 and Treg responses (140). CD8^+^ T cells are resistant to viral, bacterial infections, and even cell carcinogenesis (141), and CD8^+^ Tm cells maintain long-term antitumor activity through enhanced proliferative capacity, metabolic remodeling, and self-renewal capacity. Tm cells depend on the interaction of OXPHOS and FAO to meet their metabolic demands. (142) Overexpression of PGC-1α favors the formation of central memory in CD8 T cells but is no longer a resident memory. The overexpression of PGC-1α in CD8 T cells consistently mediates immune responses in the setting of a bacterial infection or peptide vaccination, and CD8+ T cells with elevated PGC-1α expression have been observed in mouse cancer models to provide stronger antitumor immunity. In addition, PGC-1α- overexpressing in TILs were able to retain stronger immune function and exhibited significant proliferation when the host faced the pathogen again (143). Meanwhile, Tregs play a central role in maintaining immune homeostasis so as not to “injure the innocent”, and fatty acid oxidation (FAO) acts as a major energy supply for T cells, which leads to a stronger Treg response (45). There may be a close connection between mitochondria and lipid metabolism in regulating Treg functions, thereby suggesting that immunosuppressive therapeutic strategies that regulate metabolic pathways are likely to be the future direction of autoimmune disease or cancer treatment. Although there is still a large gap in mitochondrial biogenesis on the immune function of regulatory immune cells, recent related research on Treg focuses on peroxisome proliferator-activated receptor γ (PPARγ) and its agonists. This transcription factor has long been regarded as a target protein of thiazolidinediones, and studies have found that it can enhance the immune response function of Treg by regulating fatty acid oxidation (46).

Enhancing mitochondrial biogenesis is beneficial to reinforce the immune response of relevant T cell subsets. The associated immune metabolism is affected because of defective mitochondrial biogenesis in T cells, which leads to the damage of the immune function. Meanwhile, combined immune checkpoint inhibitors could be a new strategy to address immune exhaustion in tumor microenvironments.

#### 3.1.2 Mitochondrial biogenesis and inhibitory receptor on T cells

Immune cells are regulated by several molecules that act as security brakes at desired stages of the immune responses, however, cancer cells take advantage of inhibitory immune checkpoints (ICPs) to evade tumor-specific immune responses. Recently, cytotoxic T lymphocyte-associated protein-4 (CTLA-4, CD152), programmed death-1 (PD-1, CD279) and its ligand PD-L1 (CD274, B7-H1), lymphocyte activating gene-3 (LAG-3, CD233), T cell immunoglobulin and mucin-domain containing-3 (TIM-3, CD366) have been identified as crucial targets in cancer treatment (47). Numerous studies have shown that inhibitory receptors co-expressing PD-1 and TIM-3 or PD-1 and LAG-3 on TILs are more exhausted than other TIL subsets functionally (48, 49). Analogous to chronically stimulated T cells during chronic viral infection, T cells display exhausted characteristics in the TME, such as decreased proliferation, cytokine production, and metabolic dysregulation. Whether or not T cell exhaustion can be reversed by mitochondrial biogenesis may be a new strategy for the development of immunotherapy in the future. In addition, naive T cells in lymphocytic hosts exhibit better proliferative capacity as a potential to rebuild the host immune system (50). Previte et al. investigated the role of LAG-3 in adjusting naive CD4^+^ T cell metabolism and found that defective LAG-3 adversely affected mitochondrial biogenesis and then altered Naive CD4^+^ T cell metabolism. Moreover, cells lacking the LAG3 ^-/-^CD4^+^ T cell display higher STAT5 activity and thus resistance to IL-7 deficiency, which may be considered an inhibitory pathway (51). Apart from that, it has been shown that LAG -3 is highly expressed in exhausted CD8^+^ T cells (Tex), thus leading to progressive loss of effector-effector function and memory loss after T differentiation (52). Hence, it was suggested that the recovery or reversal of the exhausted state of T cells may be associated with the combination of mitochondrial biogenesis and immune checkpoint inhibitors or costimulatory molecules highly expressed on exhausted T cells (e.g, 4-1BB) (53), the exhausted state is solved by changing the mitochondrial state to affect the metabolic function in T cell. Mitochondria biogenesis is an important step in mitochondrial quality control. When T cells are externally stimulated, mitochondria respond rapidly, activating and proliferating, and the cells differentiate into cell populations with various functions to execute the immunological effect. However, chronic pathogen stimulation can lead to T cell exhaustion. These current studies provide us with a feasible research approach to combine immune checkpoint inhibitors with co-stimulatory molecules or epigenetic modulators targeting mitochondrial biogenesis to reverse the exhausted state. Furthermore, if mitochondrial biogenesis is the “producer” in mitochondrial quality control, then fusion and fission are the “balancers” in this process.

### 3.2 Mitochondrial fusion and fission prompt T cell differentiation

Mitochondrial fission and fusion play essential roles in preserving functional mitochondria when cells experience metabolic or environmental stresses. Fusion reduces stress by mixing with partially damaged mitochondria as a complementary structure. Meanwhile, fission generates new mitochondria, contributes to efficient management with the aid of enabling the elimination of impaired mitochondria, and prompts apoptosis through excessive cellular stress (54). During T cell activation, the function of mitochondrial dynamics has gained attention, metabolic reprogramming, and differentiation. This part will describe the involvement of mitochondrial dynamics in the T cell immune response using the major proteins of mitochondrial fission and fusion as hints.

Mitochondrial biogenesis is essential for energy requirements and metabolism in tissues and is mainly coordinated through the PGC-1 family of coactivators (major regulators of mitochondrial biogenesis). These activators integrate gene expression in the nucleus and mitochondria through cascade regulation, involving the sequential activation of transcriptional regulatory proteins (55). Furthermore, these mitochondrial forming proteins that control mitochondrial fusion and fission dynamics, such as mitofusin 1 (MFN1), mitofusin 2 (MFN2), and optic nerve atrophy 1 (OPA1), enhance mitochondrial fusion and promote cellular energy production. Conversely, mitochondrial fission factor (MFF) and dynamin-related protein 1 (DRP1) are the predominant factors in mitochondrial fission (56, 57).

#### 3.2.1 OPA1—mitochondrial inner membrane fusion

Outer mitochondrial membrane fusion is induced by Mfn1 and Mfn2, meanwhile, inner membrane fusion is controlled by Opa1 (57). Each T cell subset is characterized by metabolic pathways, thus requiring precise nutrients and intracellular enzymes. Transporters of these enzymes or nutrients affect the differentiation and characterization of T cells during autoimmune responses (37). Unlike effector T cells which are more dependent on glycolysis to promote proliferation differentiation, Tm cells and T n cells rely on mitochondrial fatty acid oxidation and oxidative phosphorylation for survival and activation (21). In addition, protease Yme1L is responsible for constitutively cleaving OPA1 to form desired cristae construction (58). Moreover, SENP1 acts as an inducer of Sirt3 deacetylase activity in T cells’ mitochondria, thus resulting in a reduction in YME1L1 acetylation. Deacetylation of YME1L1 inhibits OPA1 cleavage and leads to mitochondrial fusion, thereby enhancing T cell survival and T memory cell formation (59). In addition, mitochondrial dysfunction further dampens mitochondrial fusion through inducting the OPA1 cleavage by protease OMA1 (60). Similarly, the T cell intracellular antigen (TIA1b/TIARb) and Hu antigen R (HuR) play inverse roles in modulating the expression of mitochondria-forming proteins. Mitochondrial fission and clustering are enhanced by TIA1b/TIARb, which alters the mitochondrial dynamic network and is accompanied by reduced mitochondrial respiration. In contrast, HuR activates fusion and remodel of mitochondrial cristae to augment mitochondrial respiratory activity. As a result of switching the splicing patterns of OPA1 to facilitate the production of OPA1 variant 5, TIA proteins suppress the expression of optic atrophy 1 (OPA1) protein (61). Opa1-dependent mitochondrial fusion maintains the structure of the mitochondrial cristae to promote differentiation and proliferation of T cells.

Collectively, Opa1 maintains the basic mitochondrial function and fusion morphology of T cells. Effector T (Te) cells enhance the memory properties of cells, and the anti-tumor properties of Tm cells after infection require a gradual increase in mitochondrial fusion and activation of mitochondria. Mitochondrial cristae in Tm cells are altered by mitochondrial fusion, which favors the complex association of the electron transport chain (OXPHOS) and FAO (62). In contrast, the event of the fission in Te cells makes cristae fragmented, then weakens oxidative phosphorylation efficiency and facilitates aerobic glycolysis. Hence, it is an important strategy that targets mitochondrial fusion and fission events based on the metabolic demands of T cell differentiation

#### 3.2.2 DRP1—a crucial role in metabolism reprogramming and migration

As mitochondrial fission is mediated by dynamin-related protein 1 (DRP1), a cytosolic GTPase, specific adaptor proteins are required to anchor this receptor in the mitochondrial outer membrane (MOM). Herein, self-assembling DRP1 forms a ringlike structure around mitochondria by recruiting adaptor proteins to the MOM (63, 64). The activation of Drp1 regulates the fragmentation of organelles but this protein is inactive in the cytosol, whereby it is phosphorylated on serine637 which is a suppressed phosphorylation site. In addition, its excitation signals need each the dephosphorylation of serine637 which orients Drp1 closer to the OMM, and the phosphorylation of serine616, which prompts Drp1 to implement fragmented mitochondria (65). Moreover, although the exact mechanism is unclear, Drp1 regulates the shape of mitochondrial cristae and thus mitochondrial fragmentation in an opa1-independent manner (66). Mitochondrial dynamics are not only the powerhouses of normal cells but also work as significant roles in metabolism reprogramming, migration, and clearance of damaged mitochondria, as well as in T cells and cancer cells (67, 68).

Furthermore, Drp1 is a key factor for T cell migration and proliferation. Once Drp1 is defective, defects in cell proliferation and migration are observed in developing thymocytes (69, 70). A key site for mature T cells is the T cell receptor (TCR-calcineurin signaling pathway) which triggers T cell activation (71). Calcium is an important second messenger during T cell activation at sites linked to antigen-presenting cells (APCs), whereby mitochondria shift below the plasma membrane of the immune synapse (IS). Thus, mitochondrial calcium uptake prevents calcium-dependent CRAC channel inactivation, maintains low calcium influx in the cytoplasm, and maintains an optimal T cell activation state (72) Meanwhile, calcium-dependent dephosphorylation of serine637 enhances Drp1 excitation (73). In addition, Drp1-dependent fragmented mitochondria trigger mitochondria recruitment toward the IS through the movement of fragmented mitochondria in these microtubules (70).

Meanwhile, Tn (Naive T) cells respond to antigens by activating and converting into T effector (Te) cells. Once the immune response is terminated, most Te cells will disappear, but some will remain as T memory cells, which will continue to exist for a long time. When Tm cells recognize the identical antigen, they are rapidly reactivated and proliferated into Te cells. T cells have different metabolic requirements depending on their metabolic activity, and inhibit overactivation and block inappropriate immune responses (74). Different immune phases have different metabolic demands in T cells. However, in addition to regulating mitochondrial fragmentation, the precise mechanism by which Drp1 regulates mitochondrial cristae is unclear. Moreover, the mitochondrial network is closely related to cellular metabolism. Mitochondrial fragments are always found in cells that rely on glycolytic metabolism, whereby mitochondrial fragments facilitate the breakdown of electron transport chain (ETC) complexes, thus reducing the rate of OXPHOS and more favorable to glycolysis. However, a richer network of efficiently assembled corpuscles is observed in cells with OXPHOS-based metabolism (75, 76). Drp1 decreases OXPHOS efficiency in mitochondrial debris-mediated T cells, thereby leading to the formation of ineffective mitochondrial cristae. In response to TCR stimulation, transcriptional upregulation of genes encoding glycolytic enzymes and Drp1-dependent calcium influx maintain excitation of the mTOR/cMyc pathway (62, 70). As for the Tm, the OXPHOS-based metabolism of Drp1 KO T cells affects the generation of memory-like T cells *in vivo*. Furthermore, the Drp1 deficiency-driven change in Tm was associated with increased levels of exhausted T cells *in vivo*, exhausted CD8 T cells arise from the memory precursor rather than the terminally differentiated effector CD8 T cells (77). Moreover, an important feature of T cell exhaustion is increased expression of inhibitory receptors, combined PD-1 and its ligand in activated T cells suppresses antitumor immunity by blocking stimulatory signals to T cells (78). Herein, PD-1 regulates both mTOR and ERK pathways, which are modulator of Drp1-dependent mitochondrial fission (79, 80). In addition, Drp1 and the MAPK-ERK pathway are important regulators of proper T cell migration in mitochondrial dynamic homeostasis. PD1^+^ exhausted T cells with chronic viral infection exhibit reduced mobility and lower level of ERK (80). Then, whether or not enhancing Drp1 expression effectively restore the migratory ability of PD-1-expressing exhausted T cells (77), thereby rescuing the exhausted state of Te and promoting its normal differentiation to a memory phenotype. Moreover, Drp1-mediated mitochondrial fragmentation serves as a substrate for mitochondrial autophagy. Herein, mitophagy clears the fragmented mitochondria to maintain the normal function of mitochondria in T cells.

### 3.3 Mitophagy maintains T cells homeostasis

The integrity of mitochondria is a determinant factor in cell apoptosis and necrosis (81). Cytoplasmic reactive oxygen species (ROS) activity is enhanced under antigen contact and T cell receptor (TCR) signaling is activated. However, uncontrolled ROS leads to cell necrosis, thus affecting immune cell function. On the one hand, mitochondria are the main source of ROS, and on the other hand, mitophagy eliminates ROS to main the integrity of mitochondria (82). Moreover, mitophagy plays an important role in keeping homeostasis in T cells (83). Dysfunctional mitochondria are usually accompanied by a decrease in ETC efficiency, and then cause a reduction in ATP production (84). Damaged mitochondria or accumulation of depolarized mitochondria are associated with inflammation, and cancer progression, and are the major sources of oxidants causing oxidative damage (85, 86). Meanwhile, TILs with mitochondrial defects are prone to exhaustion (87). Mitochondrial autophagy is regulated by the PTEN-induced kinase 1 (PINK)/parkin-dependent pathway, which is the main pathway that induces mitochondrial autophagy (88). Parkin promotes the degradation of MOM proteins through ubiquitination and the formation of autophagic vesicles, thereby clearing disordered mitochondria in cells.

Therefore, it is suggested that the ablation of FAM73b, a MOM protein, may trigger mitochondrial autophagy-related signaling and promote macrophage-derived IL-12 production, thereby enhancing the antitumor effects of T cells (89). T lymphocyte chemotaxis is regulated by mitochondria, and mitochondrial morphology affects T cell activation-induced cell death (AICD), where the loss of mitochondrial membrane potential drives the release of cytochrome C from mitochondrial fragments and promotes PARK2/PARKIN recruitment (90). In addition, the denitrosylase S-nitrosoglutathione reductase (GSNOR) binds to S-nitrosylation in cellular senescence and clears damaged mitochondria through autophagy (mitophagy) while regulating T cell excitation (91). Primitive T cells are forced into quiescence through secondary lymphoid organs during maturation. Moreover, CD4^+^ recent thymic emigrants (RTEs) and naive T cells had to reduce mitochondrial mass and mitochondrial reactive oxygen species *via* mitophagy (14). Decreased mitochondrial membrane potential and mitochondrial autophagy in Treg cells are associated with inhibition of Treg proliferative function, and the autophagy agonist Rapa increases the level of Treg proliferation (92). Herein, autophagy is an intracellular degradation system that plays an important role in the survival of T cells, meanwhile, mitophagy is a specific mechanism to maintain normal cellular function. Furthermore, autophagy regulates various elements of the immune system, such as pathogen clearance, antigen presentation, cytokine production, antibody response, and lymphocyte homeostasis (93). Previously, several studies reported that a lack of ATG genes (ATG5, ATG7, or ATG3) increased mitochondrial volume and reactive oxygen species (ROS) levels, whereas ATG7 is required for the survival of mature T lymphocytes (83, 94). As observed by researchers, the upregulation of autophagy adapts CD8^+^ T cells to eliminate mitochondrial depolarization, utilize functionality, and gather tissue residence, then tight MQC is imperative for T cellular homeostasis in the liver and this is offered by heightening autophagy stages. Researchers also observed that upregulation of autophagy adapts CD8^+^ T cells to eliminate depolarized mitochondria and that MQC is essential for maintaining hepatic T cell homeostasis, while this state is sustained by increasing autophagy (95). In addition, inhibition of mitophagy leads to the accumulation of ROS in cells, subsequently initiating a mitochondrial transfer to introduce healthy mitochondria to rescue cellular homeostasis may be a feasible strategy.

### 3.4 Mitochondrial transfer as a double-edged sword in T cells

**Figure 2:**
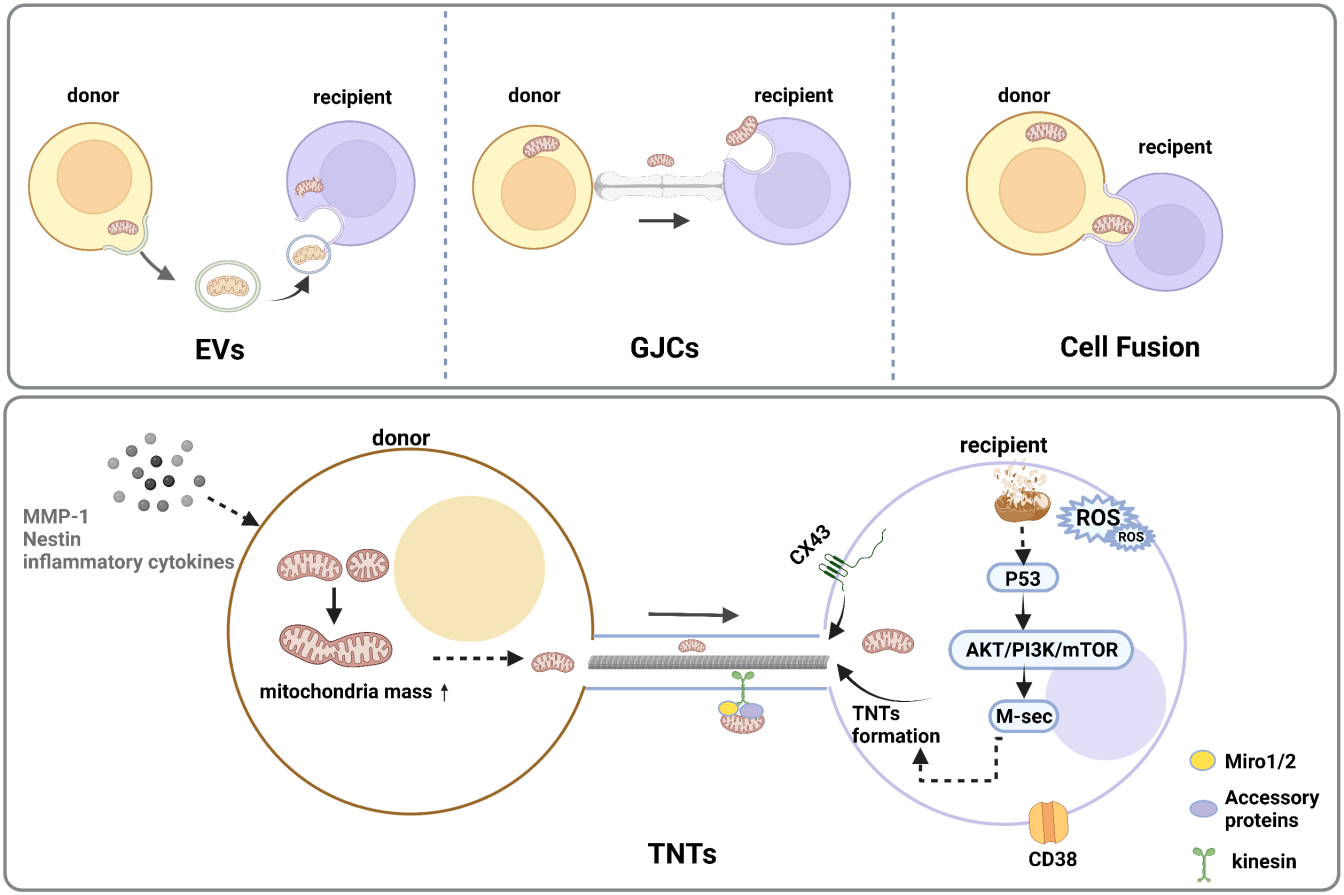
Intercellular mitochondrial transfer modes. There are four major transfer modes, such as extracellular vehicles (EVs), cell fusion, gap junction connections (GJCs), tunneling nanotube (TNTs)under the stimulation of stress reaction, inflammation response, DNA damage, and increased interstitial ROS levels. TNTs are the common type of cellular community which could transform the metabolism and functional features of the recipient cells, and the initial increase in mitochondrial mass could reveal between the donor and recipient cells. A high level of ROS in mitochondrial recipient cells can trigger activation of p53 and the downstream Akt/PI3K/mTOR axis, resulting in an overexpression of M-sec, which could enhance TNT formation.

Mitochondrial Transfer is a crucial way of intercellular communication. Herein, related studies have shown the importance of mitochondrial transfer for the regeneration of injured or infected cells and tissues (96). Functional mitochondria are transferred from the donor cells to the recipient cells with defective mitochondria, thus increasing mitochondrial mass (97–99). Moreover, there were four major transfer modes, including extracellular vehicles (EVs), tunneling nanotubes (TNTs), cell fusion, and gap junction (34). Among all transfer modes, TNTs are the main way of mitochondrial transfer, Miro 1 and 2 are two types of Rho-GTPases that connect mitochondria with other accessory proteins and move along the TNT that connects the two cells (100). (**Figure 2**) Furthermore, tumor necrosis factor α (TNFα) and NF-κB promote the formation of TNTs, which maintain cellular energy metabolism and cell survival through apoptosis, a process regulated by mitochondria (101, 102). Meanwhile, transcellular mitochondria transfer plays an important role in mitochondrial quality control. When cellular mitochondria are damaged or senescent, damaged mitochondria trigger mitochondrial fusion, fission, mitophagy, or mitochondrial biogenesis, which maintain intracellular mitochondrial homeostasis by donating normal mitochondria to surrounding cells with damaged mitochondria *via* TNTs or EVs (103, 104).

Herein, mitochondria play a major role in the progression of HCC, and both the progression and metastasis of HCC are closely related to mitochondrial mass (105). According to Otto Warburg’s hypothesis, cancer cells depend on the upregulation of glycolysis, however, inhibition of functional mitochondria induces excessive ROS accumulation and leads to apoptosis in cancer cells (25). Moreover, evading the immune system is an important strategy in tumor progression. According to Saha et al, cancer cells could hijack mitochondria from immune cells through physical nanotubes-TNTs (106). This means exhaustion of immune cells and enhancing the aggressiveness and metabolic efficiency of the tumor. Although this mitochondrial hijacking is unilateral, it has also been demonstrated that T cells additionally obtain mitochondria from other donor cells to promote cell proliferation and differentiation. Currently, mitochondrial transfer of T lymphocytes is mainly studied in autoimmune diseases, such as acute lymphoblastic leukemia (107) and graft-versus-host disease (108). Meanwhile, mesenchymal stem cells (MSCs) have significant advantages in technical extraction and efficacy and are often used as donors for mitochondrial transfer. MSCs play a key role in mitochondrial transfer by directly donating mitochondria to damaged cells and rescuing tissue degeneration caused by mitochondrial damage (109). Yuan et al. found that MSCs induced macrophages to form an anti-inflammatory phenotype and attenuated kidney injury in diabetic nephropathy (DN) mice by mitochondrial transfer (110). Furthermore, MSCs- Mito transfer activated PGC-1α-mediated mitochondrial biogenesis to resist inflammatory responses (111). Moreover, mitochondria transfer causes chemoresistance in T-ALL cells transfer mitochondria to MSCs, which would protect leukemic cells from chemotherapy (107). MSCs have important immunosuppressive properties (112). Herein, Th17 cells take up mitochondria from BM-MSCs after co-culture with BM-MSCs and reduce IL-17 production by Th17 cells, which could provide a new strategy for the treatment of chronic inflammation (113).

In brief, mitochondrial transfer is a double-edged sword. On the one hand, tumor cells deprive T cells of mitochondria through mitochondrial transfer. On the other hand, mitochondrial transfer can be used as a therapeutic target for tumors by changing the metabolic status of donors and recipients through changes in mitochondrial mass between donors and recipients (e.g, delivery of healthy mitochondria through donor cells to exhausted T cells by damaged mitochondria to compensate for impaired cellular functions).

## 4 Targeting mitochondria of T cell in hepatocellular carcinoma genesis and progression

**Figure 3:**
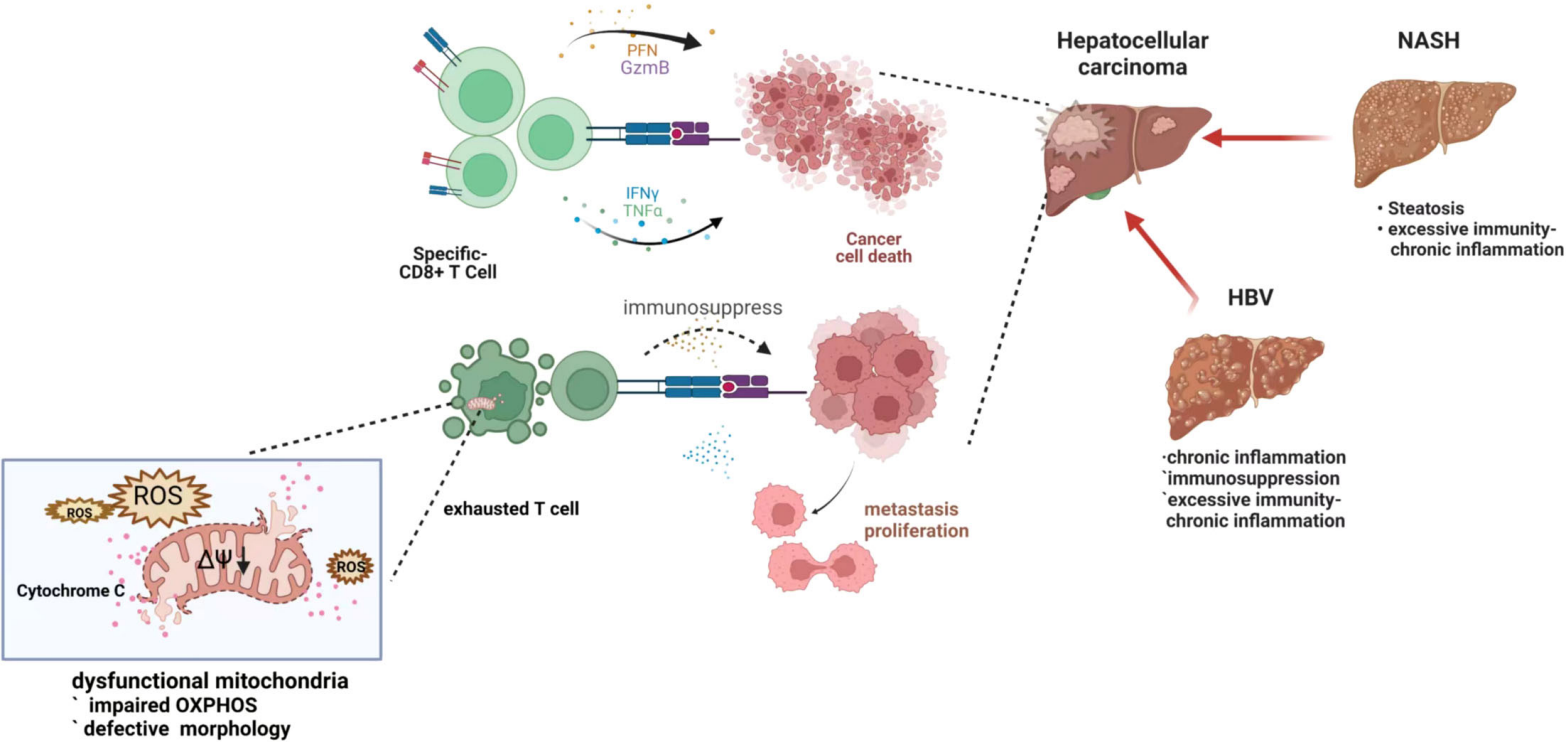
Exhausted T cell with depleted mitochondria in hepatocellular carcinoma genesis and progression. When the tumor or viral antigen is correctly recognized, the T cell immune response rapidly expands and differentiates to perform the corresponding immune function. However, under chronic antigenic stimulation, such as after HBV infection, a dysfunctional phenomenon called “exhaustion” limits the antiviral response and affects the subsequent antitumor response, which happens in immune cells with dysfunctional mitochondria. Damaged mitochondria are unable to maintain the demands of continuous immune metabolism, resulting in T cell exhaustion - loss of self-renewal and continuous differentiation.

At present, there are various clinical treatments for HCCs, such as surgical resection, liver transplantation, radiofrequency ablation, chemotherapy, and molecular targeting, however, the treatment effect on progressive HCC is still unsatisfactory (114, 115). Immune cells play a crucial role in the recognition and focus on cancer cells in immunotherapy, and immunometabolism may be an important therapeutic target by regulating mitochondria which work as a hub of the metabolism pathway. (**Figure 3**)

### 4.1 Nonalcoholic Fatty Liver Disease induced HCC

Nonalcoholic Fatty Liver Disease (NAFLD) partially evolves into NASH and eventually develops into cirrhosis and/or HCC, which is considered to be a metabolic tendency to liver cancer (116). The histological presentation ranges from the accumulation of triglycerides within the fatty liver to NASH (117). In general, the accumulation of inflammatory cells in the liver is higher in NASH than in steatosis, thereby suggesting that excitation of the immune system may contribute to the progression of fatty liver and even to HCC (118). The accruing exhausted and singularly activated CD8^+^PD1^+^ T cells in NASH-HCC has been demonstrated. However, targeting programmed death-1 (PD1)-amplified activation of intra-tumor CD8^+^PD1^+^ T cells did not result in tumor regression, thereby suggesting tumor immune surveillance was damaged and possibly because of the aberrant NASH-induced T cell activation (119).

Although the absolute number of CD4^+^ T cells is lower in NASH, there is a selective increase in Treg subpopulations (25). Herein, Treg plays an immunosuppressive role and promotes the development of tumors in the tumor microenvironment. However, Th22 and Treg cells have regulatory roles in NASH-associated HCC (NAFLD-HCC) (120). Wang et al. found neutrophil extracellular traps (NETs) were rich in the liver affected by NASH, and then verified that NETs interact with Tregs in the progression of NASH-HCC, and NETs promoted Treg differentiation by facilitating mitochondrial respiration (121). Moreover, an overactive immune response may lead to cell damage in the early stages of the disease, similar to the intense immune attack caused by HBV-HCC in earlier acute infections (122). This might be a strategy to enhance the activation of Treg NETs during NASH to HCC by increasing mitochondrial mass. Therefore, appropriately targeting of mitochondrial biogenesis in the early stages of hyper-immunity, based on the metabolic pattern on which Treg depends, enhances the immunosuppressive function of regulatory immune cells. Meanwhile, dysregulation of lipid metabolism and accumulation of lipids in the liver are important reasons for NAFLD (123). Numerous studies have reported that delaying lipid aggregation in immune cells was important for normal immune function, particularly in antitumor surveillance (124). Meanwhile, Chi et al. showed that mitochondria-induced apoptosis of CD4^+^ T lymphocytes in the liver with extensive lipid accumulation lipids free from lipid-laden hepatocytes were ingested by CD4^+^ T lymphocytes and selectively triggered cell death (125). Apart from that, excessive linoleic acid disrupts the mitochondrial function in hepatocytes and causes more oxidative damage. According to Fu et al., mitochondria isolated from HCC cells could be used to treat mice with high-fat diet-induced fatty liver. In this way, damaged mitochondria could be replaced by exogenous mitochondria, liver lipid metabolism is restored (126). Collectively, targeting mitochondrial transfer to regulate lipid uptake by CD4+ T cells may be a novel strategy for the treatment of NAFLD. Hence, further research is needed on how to target immune cell function at different stages of disease progression.

### 4.2 Hepatitis virus-induced HCC

The major risk factors for HCC are becoming increasingly related to post-sustained virological response hepatitis, active hepatitis C and B continue to drive most of the progression of HCC (116, 127). In particular, HBV causes HCC in the absence of cirrhosis similar to NASH. The transformation of HBV or HCV infection to HCC is mainly the result of long-term interaction between hepatitis virus and host hepatocytes, such as DNA integration or epigenetic dysregulation of tumor suppressor genes (128–130). The changes in the host immune system response to persistent CHB infection can be divided into five phases: immune tolerance, active, inactive, immune reactive, and HCC (131–133). During the period of hepatocarcinogenesis, CD8 T cells play the dual roles of adaptive immunity in patients with HBV-related HCC in each of the stages.

In addition, CD8 T cells are critical for HBV clearance, possibly provided by direct cytotoxicity through interferon (IFN)γ-mediated non-cytopathic lesions. Conversely, T cells are chronically exposed to antigenic stimulation during persistent chronic HBV infection (CHB), and antiviral CD8 T cells are absent, failing to mature protective T memory cells and exhaustion of HBV-specific T cells (42, 131). Considering that the effector HBV-specific CD8^+^ T cells are gradually depleted during long-term HBV infection, eventually weakening the tumor surveillance of the adaptive immune system and thus leading to immune evasion by cancer cells, thereby promoting the progression of tumorigenesis. Similarly, upregulation of transcript levels of glycolysis-related genes and impaired mitochondrial function in HCV-specific CD8^+^ T cells leads to HCV-specific T cell exhaustion (129). Schurich et al. reported mitochondrial dysfunction in hepatitis B virus-specific T cells, such as non-functional giant mitochondria and lower potential mitochondria, which inhibit chemo-oxidative phosphorylation and energy production and limit metabolic remodeling of exhausted T cells (134). Therefore, mitochondria play an important role in process of HCC. Moreover, according to Fisicaro et al, recovery of mitochondrial and antiviral CD8 functions was triggered by mitochondrion-targeted antioxidants (135). IL-12 and IL-15 can replenish exhausted T cells’ energy dependence on glycolysis and optimize cell performance by targeting mitochondrial metabolic regulation in T cells, using oxidative phosphorylation (134, 136, 137). Therefore, targeting the central role of mitochondria in metabolic reprogramming in immune cells may be a way to improve or restore effect-specific exhausted T cells in the suppressive immune environment of HCC. Mitochondria are promising therapeutic targets for chronic HBV infection by enhancing the mitochondrial feature of exhausted virus-specific CD8^+^ T cells through MQC, thereby preventing functional exhaustion.

Recent evidence suggests that despite the critical role of HBV-specific CD8^+^ T cells in the antitumor immune response, chronic inflammation mediated by overactivated CD8+ T cells promotes hepatocarcinogenesis (138). Hao et al. reported that inappropriate attack by CD8^+^ T cells plays a key role in HBsAg-driven inflammatory response and HCC tumorigenesis (139). Consequently, focusing on the different stages of disease progression and keeping the balance of the immune system are significant aspects of the treatment of HBV-related HCC.

## 5 Conclusion

Mitochondria, as important regulators of T cell physiology, play a crucial role in migration, proliferation, differentiation, and immune surveillance. Mitochondrial quality control accompanies changes in mitochondrial morphology and affects the metabolic reprogramming of cells. In addition, mitochondrial plasticity events include mitochondrial biogenesis, fusion and fission, mitophagy, and mitochondrial transfer between cells, which enable mitochondria to effectively respond to external stimulation and maintain dynamic stability in mitochondrial mass. The effector T cell (Te) metabolic mode is mainly glycolysis, however, enhanced mitochondrial biogenesis promotes metabolic adaptation and sustains immune function. Moreover, mitochondrial fission leads to the expansion of mitochondrial cristae, reducing oxidative phosphorylation efficiency and promoting aerobic glycolysis in Te cells. Te cells need to maintain the glycolytic energy supply efficiency. Furthermore, enhanced Te fusion could prompt the memory properties and anti-tumor ability of the subsequently differentiated Tm cells. Herein, the metabolic mode of Tm is mainly oxidative phosphorylation and FAO, which can effectively promote immune memory and metabolic adaptation by enhancing mitochondrial biogenesis. Apart from that, Tregs improve mitochondrial biogenesis and mitophagy, regulate FAO metabolism and prompt the immunosuppressive effect. However, in some autoimmune diseases, it is an important key point of treatment. In addition, targeting mitochondrial biogenesis may be an important approach for T cell exhaustion. The combination of immune checkpoint inhibitors and reversal of T cell exhaustion can achieve better anticancer effects, which may be applied in CAR-T and ACI. Meanwhile, in the early period of raging inflammation, it may be an effective treatment to enhance the function of Treg by targeting mitochondria. However, in the later stage, when T cells are in a state of exhaustion under long-term virus or tumor antigen stimulation, targeting mitochondria may be able to rescue the exhaustion or restore anti-tumor ability. In brief, immunotherapy for liver cancer has rapidly developed, and mitochondrial quality control of T cells may become a new research target in HCC.

With the update of immune strategies, the regulation of immune metabolism in the tumor microenvironment has become an important part. Exhausted T cells can be renewed and the anti-tumor effects were enhanced by targeting mitochondria. At present, T cell-based immunotherapy for HCC has made great progress, however, it still faces various research gaps. Herein, a few questions we might explore are whether or not liver cancer cells can also enhance their cell viability and invasiveness, and weaken the lethality and memory of T cells in the tumor microenvironment through mitochondrial hijacking. Exhausted T cells in tumor microenvironments are often accompanied by mitochondrial depletion, whether it can be delivered through healthy mitochondria *in vitro* (which can be derived from homogenous mesenchymal stem cells) to reverse the exhausted state and enhance immune efficacy. In addition, there are specific and dependent metabolic patterns in each subtype of T cell. Combining agonists or other relevant factors of mitochondrial biogenesis with immune checkpoint inhibitors (PD-1/LAG-3/TIM-3) should be considered to rescue T cells from exhaustion. Based on the stage of HCC, the mitochondria of T cells can be targeted to modulate the immune response, thereby avoiding “over-immunity” and “depletion of immunity”. Hence, gene epigenetic modulators including EnPGC-1 may be able to directly regulate mitochondrial quality control by targeting mitochondria to regulate immune metabolism is of extensive significance for T cell-centered HCC therapy.

## Author contributions

YX: Conceptualization and Writing—original draft. BG: Funding acquisition, Writing—review, and editing. XZ: Supervision, Writing—review and editing. All authors contributed to the article and approved the submitted version.

## Funding

Supported by the National Natural Science Foundation of China (Grant No.31771316). Shanghai Key Lab of Human Performance, Shanghai University of sport (NO. 11DZ2261100).

## Conflict of interest

The authors declare that the research was conducted in the absence of any commercial or financial relationships that could be construed as a potential conflict of interest.

## Publisher’s note

All claims expressed in this article are solely those of the authors and do not necessarily represent those of their affiliated organizations, or those of the publisher, the editors and the reviewers. Any product that may be evaluated in this article, or claim that may be made by its manufacturer, is not guaranteed or endorsed by the publisher.
